# Evaluating and Diagnosing Road Intersection Operation Performance Using Floating Car Data

**DOI:** 10.3390/s19102256

**Published:** 2019-05-15

**Authors:** Deqi Chen, Xuedong Yan, Feng Liu, Xiaobing Liu, Liwei Wang, Jiechao Zhang

**Affiliations:** 1 MOT Key Laboratory of Transport Industry of Big Data Application Technologies for Comprehensive Transport, School of Traffic and Transportation, Beijing Jiaotong University, Beijing 100044, China; 17114230@bjtu.edu.cn (D.C.); 16114222@bjtu.edu.cn (X.L.); 16114207@bjtu.edu.cn (L.W.); 2 Transportation Research Institute (IMOB), Hasselt University, Wetenschapspark 5, bus 6, B-3590 Diepenbeek, Belgium; feng.liu@uhasselt.be; 3 Civil Engineering Department, University of Central Florida, Orlando, FL 32816-2450, USA; zlyjsl123@knights.ucf.edu

**Keywords:** intersections, operational state evaluation, grid model, floating car data

## Abstract

Urban road intersections play an important role in deciding the total travel time and the overall travel efficiency. In this paper, an innovative traffic grid model has been proposed, which evaluates and diagnoses the traffic status and the time delay at intersections across whole urban road networks. This method is grounded on a massive amount of floating car data sampled at a rate of 3 s, and it is composed of three major parts. (1) A grid model is built to transform intersections into discrete cells, and the floating car data are matched to the grids through a simple assignment process. (2) Based on the grid model, a set of key traffic parameters (e.g., the total time delay of all the directions of the intersection and the average speed of each direction) is derived. (3) Using these parameters, intersections are evaluated and the ones with the longest traffic delays are identified. The obtained intersections are further examined in terms of the traffic flow ratio and the green time ratio as well as the difference between these two variables. Using the central area of Beijing as the case study, the potential and feasibility of the proposed method are demonstrated and the unreasonable signal timing phases are detected. The developed method can be easily transferred to other cities, making it a useful and practical tool for traffic managers to evaluate and diagnose urban signal intersections as well as to design optimal measures for reducing traffic delay and increase operation efficiency at the intersections.

## 1. Introduction

Intersections are the meeting points of pedestrians, bicycles and vehicle flow, and junctions where road users change their directions. They are also the bottlenecks of urban roads, contributing a great deal to the loss of travel time due to traffic interference, management and control [1]. Traditionally, intersection delay has been defined as the vehicle running time loss when turning or crossing intersections from upstream to downstream traffic flow [2]. Based on floating car data, Wang further characterized travel time delay as a floating car’s travel time in excess of that of user-specified free flow travel time [3]. The delay of intersections does not only affect the travel efficiency of road users but also the logic of signal control [4]. It is estimated that delay at traffic signals contributes 5–10% of all traffic delay worldwide, exemplified by the statistics that 295 million vehicle-hours of delay occurred on major roadways in the USA each year [5]. Thus, it is of striking importance for both real-time route planning and traffic control and management to accurately estimate time-dependent delay at intersections on urban road networks [6].

In the existing traffic delay research, a wide range of traffic parameters has been explored, including the total travel time [7], average waiting time [8,9], queue length [10,11,12], throughput [13], time delay [14,15], and number of stops [16,17]. The data sources mainly originated from simulation platforms or fixed road sensors (e.g., cameras and loops) [18,19,20,21]. Nevertheless, the traditional data have a number of intrinsic limitations [22,23], making it a challenge to the comprehensive evaluation of operation status at intersections based on the data. 

The development of GPS (Global Position System) technologies enables us to utilize floating cars (i.e., vehicles equipped with GPS devices) to track vehicle trajectories and collect real-time traffic data across the entire road networks. The vehicles can be regarded as ubiquitous mobile sensors, probing a city’s rhythm and pulse with high data collection efficiency [24,25,26]. Compared with the conventional data sources, the floating car data (FCD) provide three important advantages. First, real-time traffic data can be collected, and automatically sent to a processing center where information about traffic conditions is extracted [27]. Secondly, the intersections of a large part of the road network can be monitored, having a much larger coverage than sensor data by which only a limited number of areas can be observed [28]. Thirdly, via GPS-equipped vehicles, high-quality data are collected with low costs [29]. All these characteristics have led to FCD gradually becoming the mainstream data collection method in transportation research [30].

Studies have been conducted to extract traffic parameters from the FCD data, based on map-matching and Geographic Information System (GIS) techniques [31,32]. However, there are two major obstacles in applying these techniques to the analyses of intersections. First, it has been generally acknowledged that map-matching is a complicated and time consuming tool [33], making it challenging to match massive amounts of FCD across whole urban intersections [34]. Secondly, it is difficult to acquire a high quality and timely updated map which ensures the accuracy of the matches. Thus, despite the fact that FCD provide important information about traffic patterns of urban road networks [35], there has been a lack of methodologies which focus on the comprehensive evaluation of operation status at intersections using the data. To fill in this gap, in this study, a novel traffic grid model has been proposed, which is based on the FCD data but assesses the operational status at intersections more efficiently. The aim is to provide accurate estimation of traffic operation states at intersections across the whole urban road networks, and evaluate and diagnose serious problems (e.g., time delay) at the intersections. The final goal is to assist traffic managers in designing optimal measures to increase traffic operation efficiency and reduce traffic delay at urban intersections.

Compared to the existing literature, the proposed method has the following major advantages. (1) It is completely based on the data, without the aid of any additional tools such as complex map-matching and digital maps. This makes this approach particularly practical for researchers and engineers who are not familiar with the map-matching and GIS techniques. (2) This method is easily transferred to other cities for the evaluation of traffic operation at most of the signalized intersections of the urban road networks. (3) Under the grid modelling, the intersections are transformed into discrete cells, and the FCD data are matched to the grids through a simple assignment process. (4) From the constructed cell-based intersections, key traffic parameters are extracted, and intersections with the longest delay are identified and the detailed problems at these intersections are further diagnosed. The simplicity of the grid model and the parameter extraction, along with the constant input of the GPS data, makes the proposed method highly efficient in evaluating and diagnosing intersection operation in real time. 

Using the central area of Beijing as the case study, the potential and feasibility of the proposed method are demonstrated. The rest of this paper is organized as follows. Section 2 describes the FCD data and details the proposed methodology. Section 3 conducts a case study and in-depth analyses on the experimental results. Finally, Section 4 ends this paper with major conclusions and discussions for future research.

## 2. Methodology

### 2.1. Data Collection and Cleaning

The FCD data were collected by DiDi, a company offering ride-hailing services for 20 million registered taxi drivers in 380 cities in China [36]. DiDi is the largest on-demand ride service platform in China and one of the largest in the world [37]. In 2017, it served 25 million rides each day, with more than 20 billion path planning requests and data of more than 4500TB being processed.

The data were sampled at a rate of 3 s (seconds) through the GPS sensors embedded on drivers’ mobile phones, and sent to the processing center of DiDi, where traffic information was extracted from the data. The FCD data mainly consist of two parts, including GPS log files and GPS trajectories defined as follows.

**Definition** **1.** *A GPS log file* $Tr_{i}$ *represents a data record (i.e., a GPS point) transmitted from GPS sensors. The format of the log file is* <Id,t,lng,lat,v> *, where* Id *refers to the Id of the vehicle,* t *is the timestamp,* lng *and* lat *are the longitude and latitude,* v *denotes the speed of the vehicle.*

**Definition** **2.** *A GPS trajectory* $Tra_{k}$ *is a sequence of GPS log files, i.e.,* $Tra_{k}=Tr_{1}\to Tr_{2}\to \cdots Tr_{m}\to \cdots Tr_{n}$ *, with* $Tr_{m}=<Id_{m},t_{m},lng_{m},lat_{m},v_{m}>$ *, where the time stamps in the sequence strictly increase.*

The obtained GPS trajectory data were saved in txt files, and the variables and their attributes are listed in Table 1. 

Raw GPS data may contain errors caused either by blockage of GPS signals or by hardware/software bugs during the data collection and transmission process [38]. In order to provide good quality of data and ensure accuracy of the derived results, it is important to detect and remove the wrong records that are incompatible with the physical phenomena of traffic. The data cleaning process is carried out according to the following two steps.

***Step1:*** Remove the data that are beyond the range of the traffic analysis region.

***Step2:*** Remove the data in which the corresponding vehicle speeds are out of the range of 0–90 km/h.

### 2.2. The Grid Model

**Figure 1:**
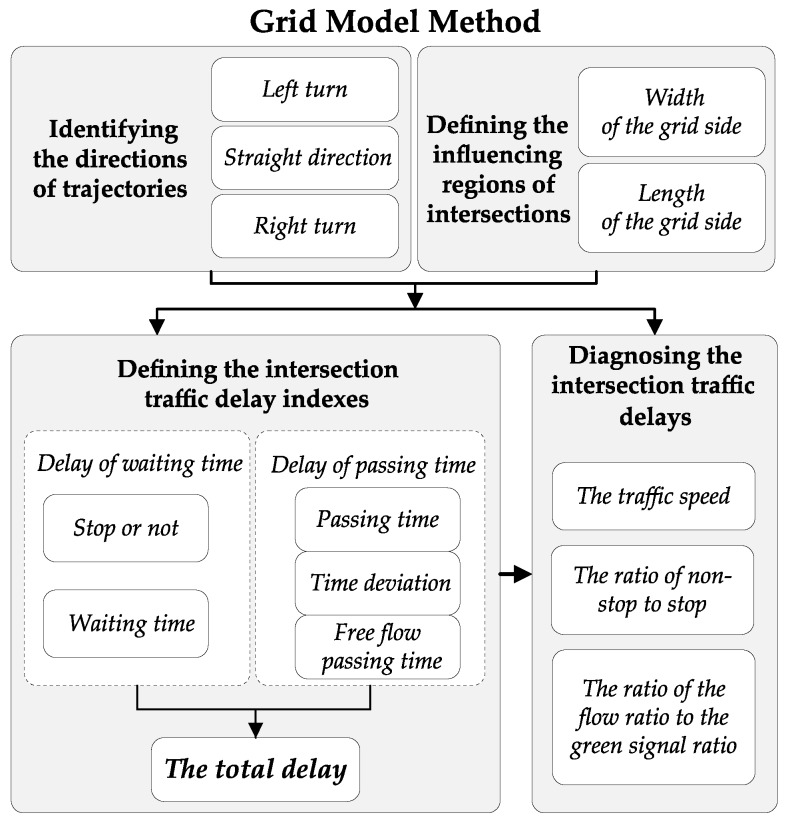
The flowchart of the grid model method.

The traffic grid model is based on the City Management Grid Modelling Theory [39]. In this technique, a management area can be divided into disjoint grids with the same or different sizes. The facilities within the grids are then managed and dispatched by different categories (such as different types of civil facilities) in order to improve the management effectiveness. Figure 1 shows the flowchart of the grid model method that we applied in this study. Firstly, the procedures of identifying the directions of trajectories and defining the influential regions of intersections are used to extract the key traffic parameters from floating car data, which are further used for the intersection traffic delay index calculation and intersection of traffic delay diagnosis.

#### 2.2.1. Identifying the Directions of Trajectories

**Figure 2:**
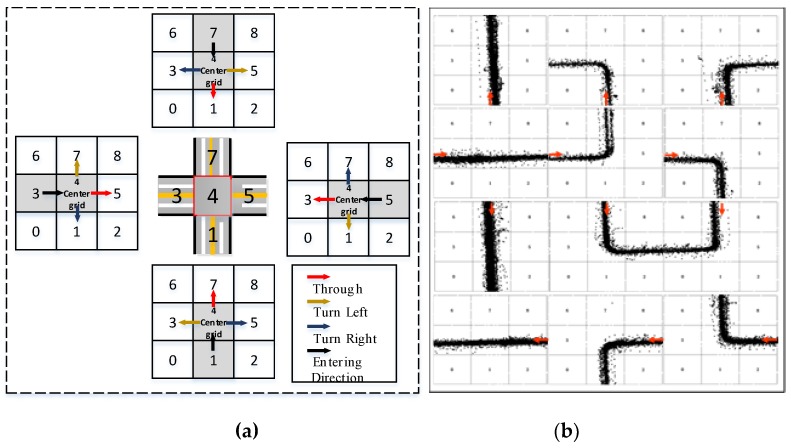
A basic grid network: (**a**) searching direction in mapping an intersection to a grid network, (**b**) the result of the direction of trajectories identified.

Once the intersections are split into grids, the points of each trajectory are then mapped to the grids (see Figure 2a). According to the specific order of the Grid ID (i.e., from 0 to 8) of the first entrygrid and that of the last exit grid that vehicles pass through, the direction of the trajectory is subsequently identified. Thus, intersections are reconstructed into grid-based ones, as displayed in Figure 2b. The trajectory data along with the identified directions can be utilized to extract the traffic performance parameters at the intersections.

#### 2.2.2. Defining the Influential Regions of Intersections

In the current study, we divide the area of each road intersection into grids with an unfixed size. In constructing such grids, two important parameters need to be specified: one is the range of the intersection area, and the other is the size of each grid. Different ranges of intersections have been adopted in previous studies. For instance, Sun investigated Beijing’s road networks and defined the range as 160 m [40]. Zhang et al. considered the dynamic spatial distribution characteristics of floating cars, and set the range as 200 m, which started at the center of an intersection and extended both upstream and downstream by 100 m [41]. Choosing an appropriate intersection range is essential to distinguish different intersections, and ensure enough data in each intersection as well as in each cell of the intersection. If the range is too large, it may contain multiple intersections, and the traffic conditions between different intersections cannot be differentiated. On the contrary, if the range is too small and cannot cover an intersection adequately, the FCD data samples would be insufficient for some cells of the intersection, leading to the inaccurate calculations of travel speed and vehicle positions.

**Figure 3:**
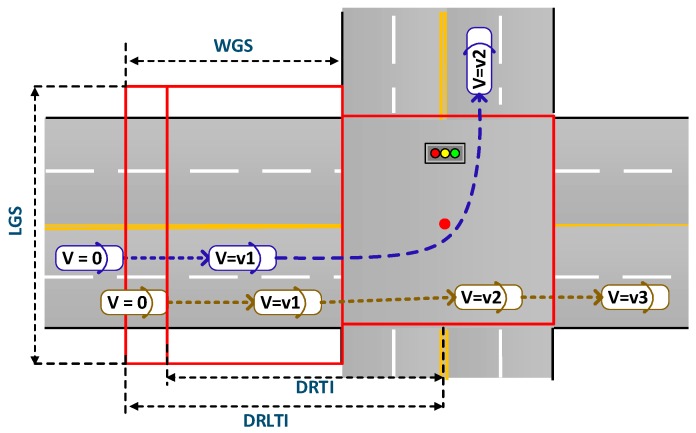
The region of an intersection defined by the grid model.

Given the above consideration, we empirically estimate the range of intersections using the FCD data, which should cover the upstream spaces of intersections from the center points of the corresponding intersections to furthest queuing points of vehicles at intersections. According to the spatial-temporal FCD’ distributions, there will be different affected areas in different directions of intersections: the defined region of straight-through traffic (i.e., DRTI), the defined region of left-turn traffic (i.e., DRLTI), as shown in Figure 3. It is noted that the width of the grid side (i.e., WGS) was influenced by DRLTI and DRTI, whose boundaries are the maximum stop distances for trajectories, where the velocity of the position is 0m/s (i.e., V = 0). Then, we choose the 95th percentile of the distance of vehicles at the intersection between the stop point and the center point of the intersection as the defined region of different directions. The length of the grid side (i.e., LGS) should make sure that the FCD data are fully matched to the grids.

#### 2.2.3. Defining the Intersection Traffic Delay Indexes

**Figure 4:**
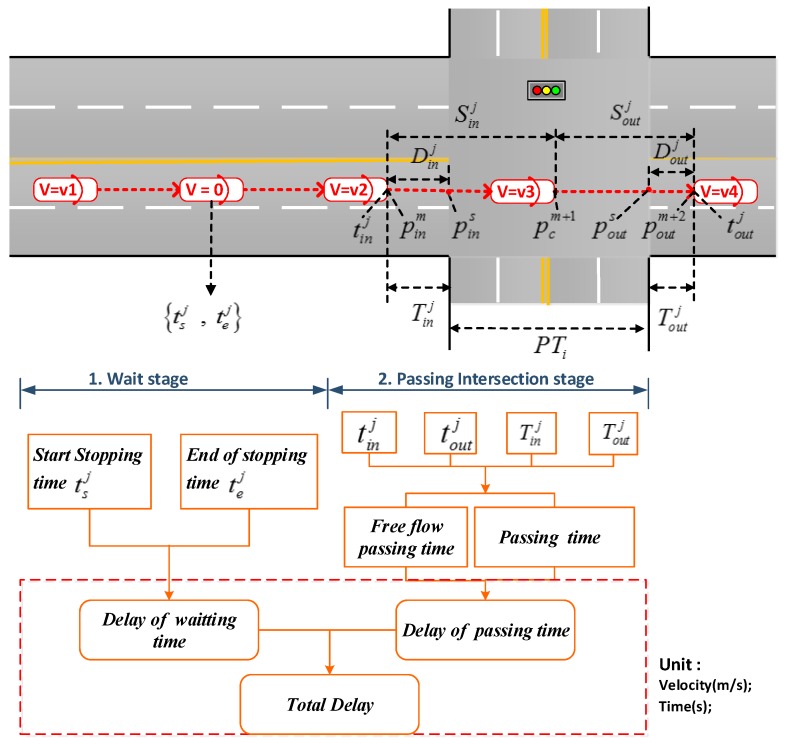
The total delay framework.

Each trajectory j of FCD passing the intersection of the process has two stages: waiting stage and passing stage; non-stop trajectories do not involve the waiting stage. For each intersection, the corresponding total delay (i.e., $TD_{j}$) can be divided into two aspects: delay of waiting time (i.e., $DWT_{j}$) and delay of passing time (i.e., $DPT_{j}$), respectively, as shown in Figure 4.

For each intersection, two key traffic parameters are derived, including the vehicle passing time (i.e., $PT_{i}$) through each direction i of the intersection and the waiting time delay (i.e., $WT_{i}$) of the direction i. To this end, the following variables are defined in Table 2.

Three time slices (i.e., 5 min, 15 min and 30 min), are considered for the computations of the traffic parameters. The delay of go-through the intersection direction *i* is computed based on Formula (1). The free flow passing time (i.e., $TF_{i}$) in the direction *i* is assumed as the 5th percentile of the distribution of all the passing time derived from the FCD data, as suggested by Brown et al. [42].

(1) $$DPT_{i}=PT_{i}-TF_{i}=\frac{\sum_{j=1}^{N_{t}}\left(t_{out}^{j}-t_{in}^{j}-T_{in}^{j}-T_{out}^{j}\right)}{N_{t}}-TF_{i},\;t=5\;\min,15\;\min,30\;\min$$

Because most of the trajectory points arriving at and leaving the intersection do not exactly match the boundary of the intersection, the time deviation is produced. Time deviations of the entry and exit intersection can be calculated by $D_{in}^{j}$, $S_{in}^{j}$, $D_{out}^{j}$, $S_{out}^{j}$ and 3 s time interval. As is shown in Figure 4, $D_{in}^{j}$ is defined as the distance between $p_{in}^{s}\left(Lat_{in}^{s},Lng_{in}^{s}\right)$ and $p_{in}^{m}\left(Lat_{in}^{m},Lng_{in}^{m}\right)$, $S_{in}^{j}$ is defined as the distance between $p_{in}^{m}\left(Lat_{in}^{m},Lng_{in}^{m}\right)$ and $p_{c}^{m+1}\left(Lat_{in}^{m+1},Lng_{in}^{m+1}\right)$, $D_{out}^{j}$ is defined as the distance between $p_{out}^{s}\left(Lat_{out}^{s},Lng_{out}^{s}\right)$ and $p_{out}^{m+2}\left(Lat_{out}^{m+2},Lng_{out}^{m+2}\right)$, and $S_{out}^{j}$ is defined as the distance between $p_{out}^{m+2}\left(Lat_{out}^{m+2},Lng_{out}^{m+2}\right)$ and $p_{c}^{m+1}\left(Lat_{in}^{m+1},Lng_{in}^{m+1}\right)$, where the intersection point of the trajectory and boundary is *s*, and the points *m*, *m* + 1, *m* + 2 in trajectory *j*. Therefore, the time deviations of the entry intersection and exit intersection ($T_{in}^{j}$, $T_{out}^{j}$) can be obtained by Formula (2) and (3).

(2) $$T_{in}^{j}=3\frac{D_{in}^{j}}{S_{in}^{j}}\;=3\frac{\mathrm{Arccos}(\sin(Lat_{in}^{m})\sin(Lat_{in}^{s})\cos(Lng_{in}^{s}-Lng_{in}^{m})+\cos(Lat_{in}^{s})\cos(Lat_{in}^{m}))}{\mathrm{Arccos}(\sin(Lat_{in}^{m})\sin(Lat_{in}^{m+1})\cos(Lng_{in}^{m+1}-Lng_{in}^{m})+\cos(Lat_{in}^{m+1})\cos(Lat_{in}^{m}))}$$

(3) $$T_{out}^{j}=3\frac{D_{out}^{j}}{S_{out}^{j}}=3\frac{\mathrm{Arccos}(\sin(Lat_{out}^{m+2})\sin(Lat_{out}^{s})\cos(Lng_{out}^{s}-Lng_{out}^{m+2})+\cos(Lat_{out}^{s})\cos(Lat_{out}^{m+2}))}{\mathrm{Arccos}(\sin(Lat_{out}^{m+2})\sin(Lat_{in}^{m+1})\cos(Lng_{out}^{m+2}-Lng_{in}^{m+1})+\cos(Lat_{in}^{m+1})\cos(Lat_{out}^{m+2}))}$$

The delay of waiting time (DWT) can be computed by Formula (4), in which the non-stop trajectory *j* leads to φ=0, otherwise φ=1.

(4) $$DWT_{i}=\frac{\sum_{j=1}^{N_{t}}\varphi \left(t_{e}^{j}-t_{s}^{j}\right)}{N_{t}},\;t=5\min,\;15\min,\;30\min$$

The traffic delay ($TD_{i}$) in direction *i* and the total delay (TD) over all the directions are computed as Formula (5).

(5) $$TD=\sum_{i=1}^{8}TD_{i}=\sum_{i=1}^{8}\left(DWT_{i}+DPT_{i}\right)$$

#### 2.2.4. Diagnosing the Intersection Traffic Delay

There are two main reasons leading to frequent delays at signalized intersections: one is the unreasonable timing of signal lights, and the other is the large traffic volume which exceeds the traffic capacity of the intersections [43,44]. The diagnosis method of traffic delay at intersections is comprised of three steps. In the first step, the delay of each single intersection is computed according to Formula (1), using the time slices of 5 min, 15 min and 30 min. Based on the obtained results, the pattern of the delay for each intersection throughout the day, particularly during the rush hours, can be analyzed. Furthermore, the delays across all the intersections are sorted, and the ones with the worst performance are identified. In the second step, detailed traffic operation at the intersections with the serious delay problems is examined. Variables, including the time delay in different directions, the average speed, the ratio between the number of non-stop trajectories and that of stop trajectories, are investigated. In the third step, the delay problems are further diagnosed based on the comparison between the flow ratio and the signal split ratio of the effective green time. According to the above diagnostic method, parameters are defined as shown in Table 3. It should be noted that $Q_{i}$, the observed number of floating vehicles passing through the intersection, is used rather than the real traffic volume at the intersection. Although the sampling rate of FCD may change in each signal cycle, historical analyses with multiple days’ data can steadily reflect the traffic volume of direction *i* of intersections and their changing patterns.

The ratio of non-stop to stop is defined as the ratio between the number of non-stop trajectories and that of stop trajectories in direction *i* (See Formula (6)). It reflects the traffic condition and the efficiency of signal timing in the corresponding direction of the intersection.

(6) $$\tau_{i}=\frac{N_{s}}{N_{ns}}$$

The red light time per direction in a signal cycle is estimated using the first stop vehicles’ trajectories that experience only one cycle at the intersections. Then, we choose the 95th percentile of the stop times of the vehicles at the intersection as the red light time. The 95th percentile of the stop times is selected because the floating car driver may have an abnormally long reaction time when the light turns green.

The vector $RT_{\phi }$ for the red light time of ϕ phase is defined as:

(7) $$RT_{\phi }=r_{lj,\phi }-r_{fj,\varphi }$$

The effective green light time is defined as:

(8) $$g_{e,\phi }=C-RT_{\phi }$$

The green time ratio is defined as the ratio between the effective green light time and a signal cycle:

(9) $$\lambda_{\phi }=\frac{g_{e,\phi }}{C}=1-\frac{(n-1)RT_{\phi }}{\sum_{k=1}^{n}RT_{\phi }}$$

The traffic flow in different directions varies; the weight of the traffic flow in the direction *i* is:

(10) $$\omega_{i,\phi }=\frac{Q_{i,\phi }}{\sum_{i=1}^{8}Q_{i,\phi }}$$

$\eta_{i,\phi }$ is defined as the ratio between the flow ratio (i.e., $\omega_{i,\phi }$) and the green signal ratio (i.e., $\lambda_{i,\phi }$) in the direction *i* of each phase ϕ:

(11) $$\eta_{i,\phi }=\frac{\omega_{i,\phi }}{\lambda_{i,\phi }}$$

Using $\eta_{i,\phi }$, we can diagnose problems of intersection delay caused by signal timing design. If the $\eta_{i,\phi }>1$ (i.e., $\omega_{i,\phi }$ > $\lambda_{i,\phi }$), the effective green time of the phase ϕ is not enough for vehicles in a signal cycle to pass the intersection. If $\eta_{i,\phi }<1$ (i.e., $\omega_{i,\phi }$ < $\lambda_{i,\phi }$), the effective green time of the phase ϕ is underutilized.

## 3. The Empirical Case Study in Beijing

**Figure 5:**
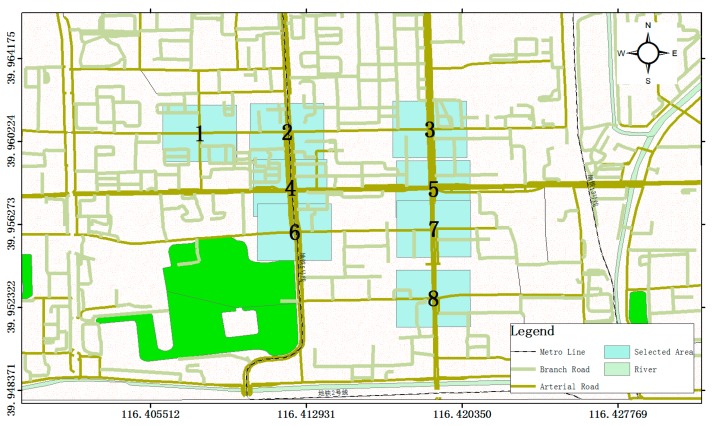
The Case of the Selected Area.

The data used in this case study include 2,712,744 floating cars’ trajectories (i.e., 35.7 GB in size) collected from 1 to 15 in August 2017, in the central area of Beijing with longitudes and latitudes as follows: <116.399450, 116.431700> and <39.947934, 39.966344>. In the study area, eight intersections were selected, namely, NO.1–NO.8 (See Figure 5), and they cover various types of intersections, including the intersecting of both arterial and branch roads (i.e., NO.2, NO.3, NO.6, NO.7, NO.8), only arterial roads (i.e., NO.4 and NO.5), and only branch roads (i.e., NO.1).

**Figure 6:**
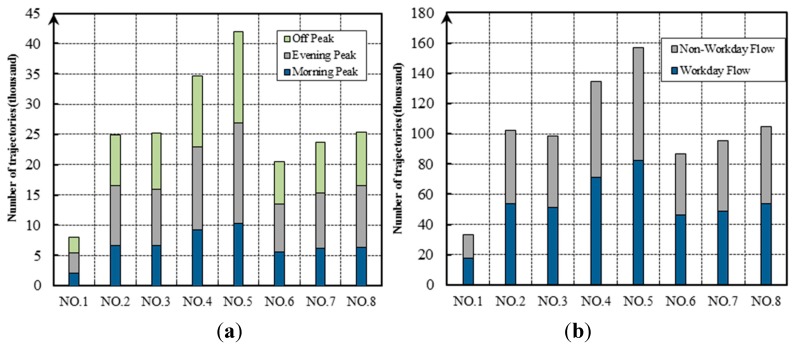
(**a**) Hourly and (**b**) daily distributions of traffic flow at the selected intersections. The Morning Peak (7:00–10:00) The Evening Peak (17:00–19:00) The off Peak (14:00–16:00).

Figure 6 shows the distributions of the averages of hourly and daily traffic flows across all the selected intersections. It was noted that the traffic flow in the evening peak hour (i.e., 17:00–19:00) was larger than in other periods, including the morning rush hour (i.e., 7:00–10:00) and the off-peak period (e.g., 14:00–16:00) (See Figure 6a); while during the week, the flow was larger on workdays than on non-workdays (See Figure 6b).

### 3.1. The Intersections’ Total Delay

**Figure 7:**
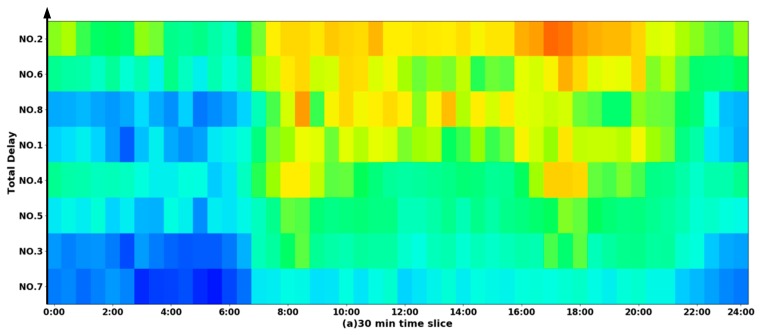

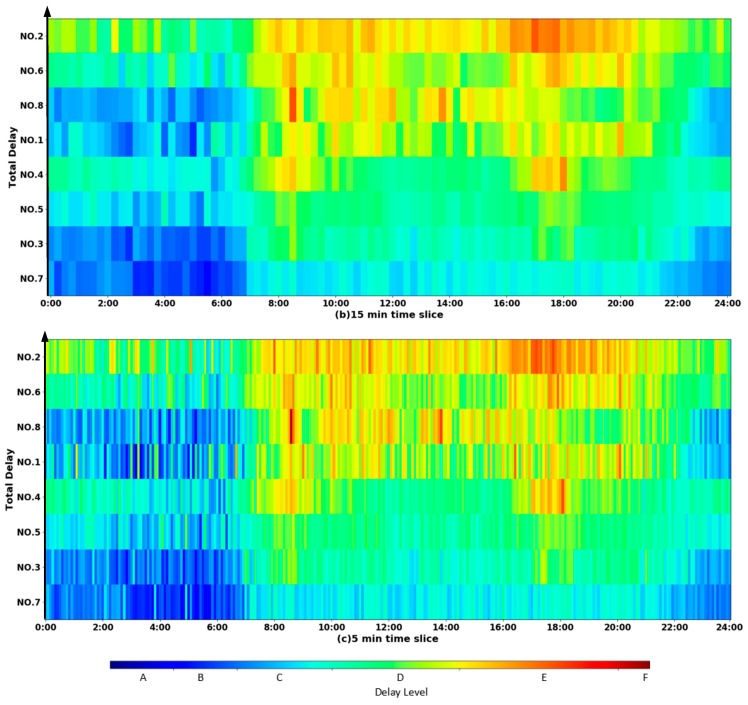
Intersection delay.

To analyze the total delay at each of the intersections, we construct the spatial-temporal diagrams with the time slices of 30 min (Figure 7a), 15 min (Figure 7b) and 5 min (Figure 7c), respectively. For each intersection (indicated by the y-axis), the different levels of delay along the different times of the day (indicated by the x-axis) are described using a color scheme, with the darkest blue referring to the shortest delay (i.e., Level A) and the darkest red to the longest one (i.e., Level F). Moreover, all eight intersections are sorted in terms of their total delay, and this order is indicated with the arrow on the left side of these figures. It was observed that the delay at the NO.2 intersection was the longest, particularly during the morning and evening rush hours. However, the delay at the NO.7 intersection was the shortest, which could be due to the fact that it is forbidden to make a left turn at this intersection. 

**Figure 8:**
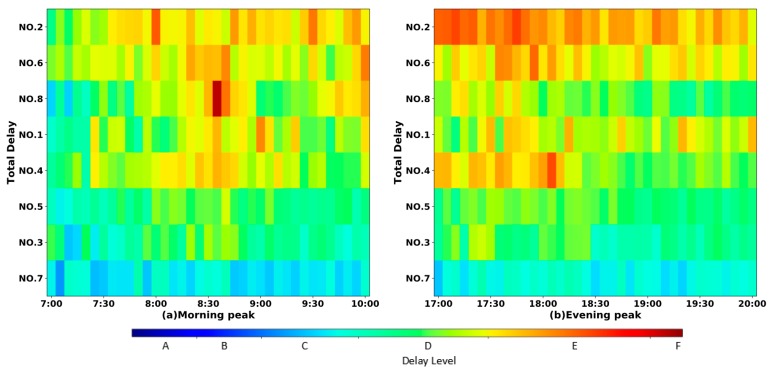
The intersection delay of the morning peak and evening peak.

Figure 8 further details of the distribution of the delay with the slice of 5 min during the two peak periods. It shows that the delay at intersection NO.2 reached the longest at 8:00–9:30 in the morning, and at 17:00–19:45 in the evening.

### 3.2. The Traffic Operation States for Individual Intersections

Apart from the total delay, other major traffic parameters including the traffic flow, average velocity and delay in each direction of each of the intersections are further investigated. In the following, the intersection with the longest delay among these selected ones, i.e., NO.2. intersection, is analyzed. The same process can be applied to any of the remaining intersections.

#### 3.2.1. The Traffic Delay

**Figure 9:**
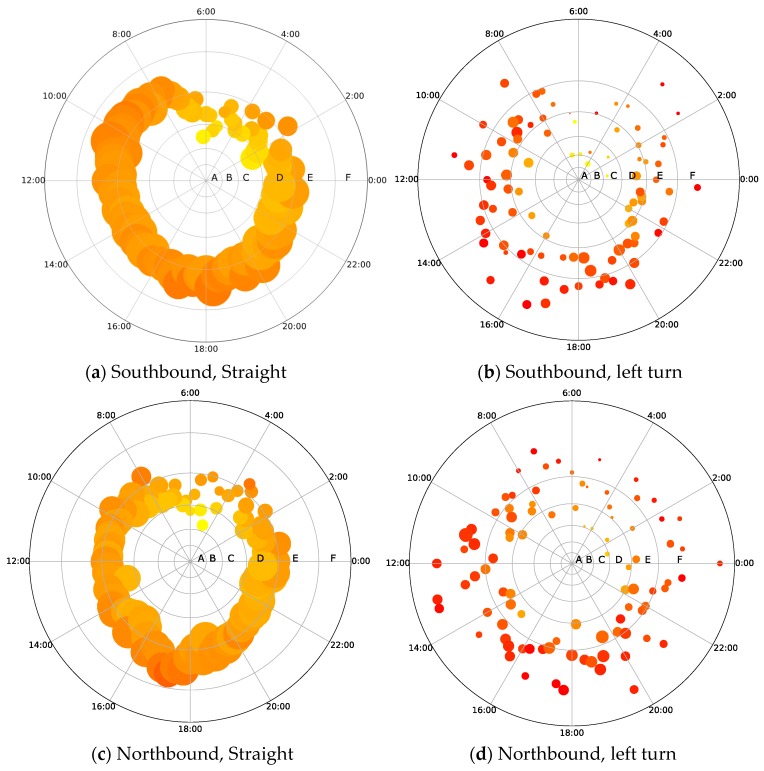

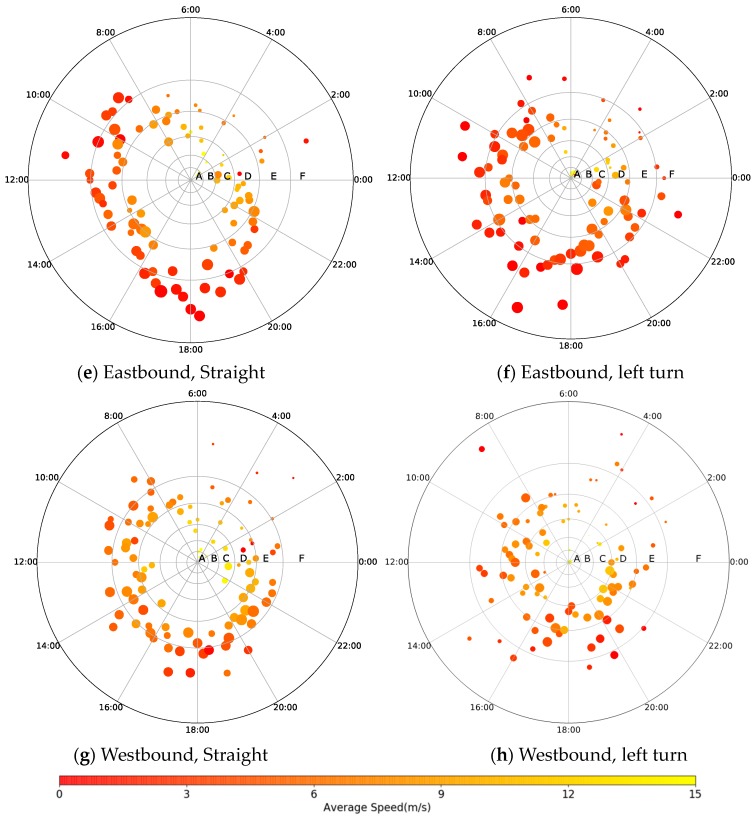
Intersection internal evaluation.

Figure 9 describes the distribution of these three major parameters across different times of working days at the intersection, using the time slice of 15 min. Each point depicts a vector of three values of these parameters, including the traffic volume (i.e., represented by the size of the point), the average velocity (m/s) (i.e., represented by the color of the point), and the delay level (i.e., represented by the radius of the point at the polar coordinates). A point that is further from the origin of the coordinates and has a larger size and red color refers to a traffic state featured with more traffic volume, lower speeds, and longer delay.

From Figure 9, it was noted that the traffic flows in the south- and northbound straight directions (See Figure 9a,c) were higher than those of the east- and westbound straight directions (See Figure 9e,g). When the conditions between the south- and northbound directions were compared, the northbound traffic flow was lower, its traffic speed was slower, and therefore the corresponding traffic delay was shorter. 

From Figure 9b,d, it was found that the distributions of the traffic flow and average velocity for the southbound and northbound left turn directions are similar. However, the southbound left turn had the longest delay during 16:00–20:00, while the northbound left turn suffered the worst delay problem at 10:00–14:00.

Figure 9e–h further show that the eastbound traffic average speeds for both the straight and left turn directions were much slower than the westbound average speed. Consequently, the delays in the eastbound directions were longer, particularly during the morning and evening rush hours. 

In summary, Table 4 lists all of the key traffic performance parameters of the eight critical directions for the intersection.

#### 3.2.2. The Traffic Speed

**Figure 10:**
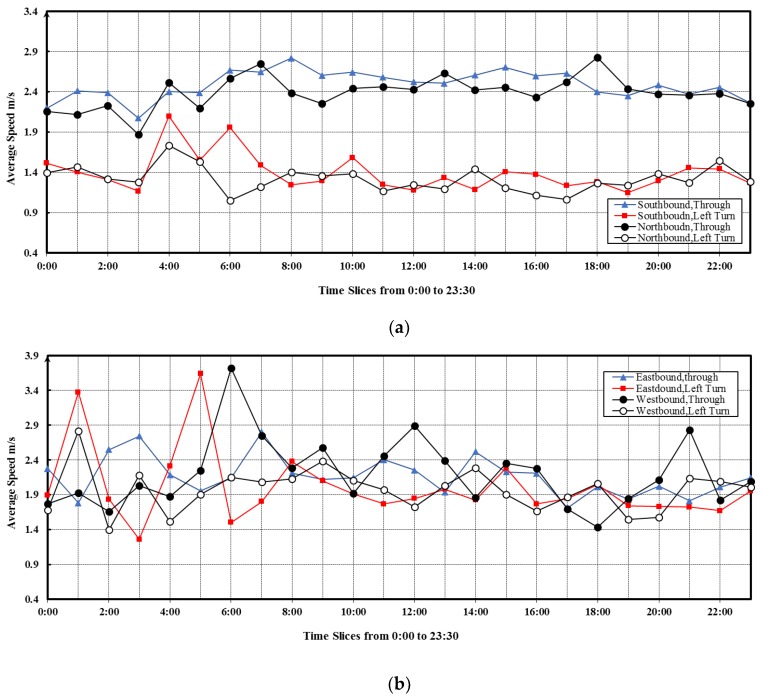
Average speed through the intersection (**a**) the direction of south and north (**b**) the direction of east and west.

For further analysis of the average speed, we compared the distribution of the average speed among different directions of the intersection, as depicted in Figure 10. A clear distinction was observed in the speeds between the straight and left turn directions in each of the southern or northern routes (See Figure 10a). In contrast, there was no large difference between the speeds of the straight and left turn directions for each of the eastern or western routes (See Figure 10b). This suggests that there would be a protected left-turn phase for the south- and northbound flows, causing little interference to the go-straight traffic. However, there was no protected left-turn designed for the east- and westbound flows. 

#### 3.2.3. The Ratio of Non-Stop FCD to Stop FCD

**Figure 11:**
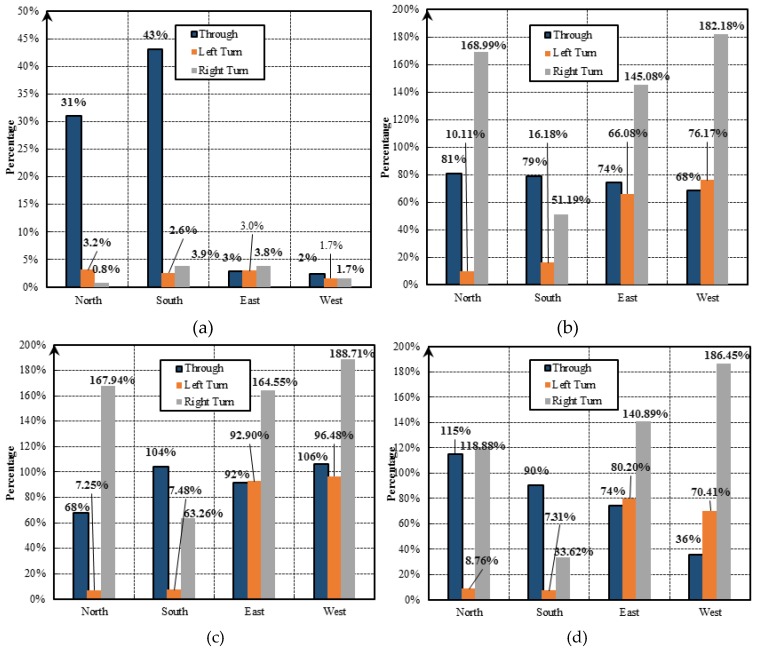
The ratio of the number of non-stop cars to the number of stops: (**a**) The percentage of the number of stops in different directions within NO.2 intersection; (**b**) the ratio of the number of non-stop cars to the number of stops; (**c**) The $\tau_{i}$ in different directions at NO.2 intersection during the morning peak (7:00–10:00); (**d**) The $\tau_{i}$ in different directions at NO.2 intersection during the evening peak hours (17:00–19:00).

Figure 11a shows the total number of stop trajectories over the entire day across different directions of the intersection, indicating that the numbers in the south- and northbound straight directions (i.e., 33% and 31%, respectively) were much higher than those in the east- and westbound straight directions (i.e., 3% and 2%, respectively). On the contrary, the numbers of stop trajectories in the left turn of all the north-, south-, east- and westbound directions were similar (i.e., 3.2%, 2.6%, 3.0% and 1.7%, respectively). However, according to the ratio ($\tau_{i}$) between the number of non-stop trajectories and that of stop ones, as described in Figure 11b, $\tau_{i}$ was much lower in the left turn of the north- and southbound directions (i.e., 10.11% and 16.18%, respectively) than that of the east- and westbound directions (i.e., 66.08% and 76.18%, respectively). This indicates that the number of non-stop trajectories was smaller and the traffic conditions were worse in the left turn of the north- and southbound directions than in the east- and westbound directions.

Figure 11c,d further depict the values of $\tau_{i}$ during the morning and evening rush hours, respectively. It was observed that $\tau_{i}$ in the left turn of the north- and southbound directions in these two periods (i.e., 7.25% and 7.48% in the morning and 8.76% and 7.31% in the evening) was lower than that for the entire days (i.e., 10.11% and 16.18% as shown in Figure 11b). This suggests that in the peak period, the unreasonable timing of signal lights would be unable to match the increasing of traffic flow. Moreover, $\tau_{i}$ in the northbound straight direction during the morning peak (i.e., 68%) was lower than that in the evening peak (i.e., 115%). Meanwhile, an opposite trend was observed in the southbound straight direction, in which $\tau_{i}$ is higher in the morning (i.e., 104%) and lower in the evening (i.e., 90%). This indicates the existence of a tidal phenomenon in the traffic flow between the north- and southbound straight directions.

In summary, Table 5 lists all of the number of stops and the ratio of the number of non-stop cars to the number of stop ones of the eight critical directions for the intersection.

### 3.3. Diagnosis of the Delay Problems for Individual Intersections

**Figure 12:**
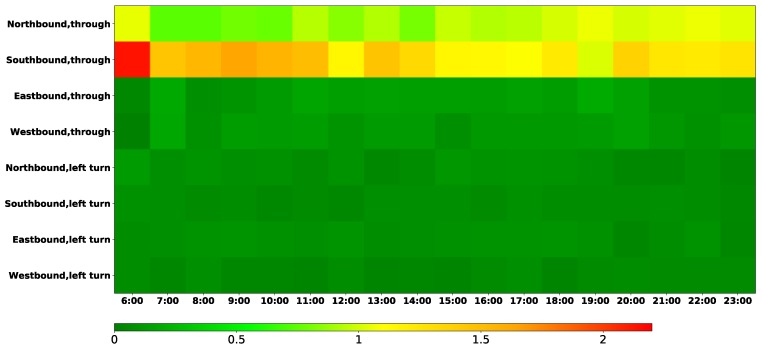
Comparison of the $\eta_{i,\phi }$ in each direction.

Figure 12 characterizes the distribution of $\eta_{i,\phi }$ (i.e., the ratio between the flow ratio and the green time ratio) over different directions of the intersection in each of the phases. It should be noted that $\eta_{i,\phi }$ values of the north- and southbound straight directions were greater than those in other directions. Table 6 further lists the specific parameter values of $\omega_{i,\phi }$ and $\eta_{i,\phi }$ in each phase of each time scheme. It indicates that the minimum $\eta_{i,\phi }$ values in north- and southbound straight directions phase were greater than that in the other phases in all the timing schemes. This implies that the effective green time of phase of the north- and southbound straight direction would be much shorter than it should be, which leads to the longer delay in the corresponding directions of the intersection. 

## 4. Discussion and Conclusions

In this study, a novel traffic grid algorithm was proposed, which is based on the FCD data but makes the map-matching process for signalized intersections more efficient. Furthermore, it provided an accurate estimation of traffic operation states at the intersections and an efficient traffic performance evaluation for signalized intersections at the network level, and can be applied to diagnose the specific problems for individual intersections.

The traditional data-collecting devices cover only fixed points of the traffic network, such as loop detectors, which are impossible to produce reliable information about travel time within the network [45]. In comparison, based on the massive amount of FCD data that are highly spatial-temporal detailed, their 3s sampling rate guarantees the accurate estimation of these intersections’ operation parameters. This study selected three kinds of time dimensions (i.e., 5 min, 15 min, 30 min) as the time slices to describe the intersections’ operation states. As for the traffic analysis region of defined intersection, Zhang et al. defined a fixed one with a 200 m length from the center of an intersection to a 100 m distance from upstream and downstream directions [41]. The fixed analysis region may cause an inaccurate estimation of the traffic parameters because the upstream queue lengths might be frequently beyond the 100 m limit, especially for large-scale intersections. In this study, the differences of intersections’ configurations and the spatial-temporal FCD’ distributions were considered, and the region sizes are variable corresponding to the actual traffic performance, which guaranteed the assurance of the veracity of the arithmetic.

In previous studies, only one or two traffic parameters (e.g., delay and stop rate) were used for intersection performance analyses, which were insufficient to explore the traffic operation performance deeply [46]. Using the radar figures (e.g., Figure 9), we combined the three traffic parameters (i.e., time delay, traffic flow, average speed) to estimate the traffic intersection operation state and evaluate the intersections’ levels of service. Thus, the intersections’ operation performances in the network can be sorted, and the ones with the worst performance can be identified. The intersections with the serious delay problems can be further investigated in terms of the traffic condition in different directions. It was found that there was a significant difference in the average speed between the straight and the left turn directions when unprotected left-turn phases were applied, which is consistent with the studies of Angell et al. and Wang et al. [47,48]. In addition, the ratio of non-stop to stop FCD ($\tau_{i}$) also exhibits variations across different times of the day and in different directions. Particularly, a tidal phenomenon of traffic flow during the morning and evening peak hours was discovered. Traffic engineers should take the tidal phenomenon into consideration when designing traffic signal control strategies. For the individual intersections, the traffic delay problem can be further diagnosed based on the parameter $\eta_{i,\phi }$. The value of the parameter is either larger or smaller than one, indicating imbalanced signal phasing design issues for the intersections, which can be utilized as an efficient tool for individual intersections’ signal phase readjustment.

Owing to unavailability of loop detector data for the case study, we could not examine the floating cars’ penetration ratio in the total traffic. However, this study focuses on the traffic signal intersection performance evaluation. The huge numbers of FCD can sufficiently reflect the historical traffic conditions and accurately provide travel speed information. Therefore, the comparative results of levels of service between the intersections in the traffic networks and the diagnoses of signal phasing design issues for individual intersections should be reliable. In future work, if the loop detector data are available, it is suggested to consider the floating cars’ penetration ratio in total traffic in order to provide more detailed methods for the real-time cases that the samples of FCD are insufficient during some cycles.

## Figures and Tables

**Table 1:** The detailed data attributes of the FCD system.

Characteristic	Field Name	Field Type	Field Description
Id	Terminal ID	String	6 bytes characters, marking each vehicle
t	GPS time stamp	Timestamp	Accurate to second
lng	Longitude	Floating	Accurate to six decimal places
lat	Latitude	Floating	Accurate to six decimal places
v	Vehicle Speed	Integer	Kilometer per hour

**Table 2:** The parameter definition of intersection traffic delay.

Parameter Notation	Definition
$t_{s}^{j}$	Time stamp of the point of the trajectory *j* starting stop
$t_{e}^{j}$	Time stamp of the point of the trajectory *j* ending stop
$t_{in}^{j}$	Time stamp of the point of the trajectory *j* enter intersection
$t_{out}^{j}$	Time stamp of the point of the trajectory *j* exit intersection
$T_{in}^{j}$	Time deviation of the trajectory *j* enter intersection
$T_{out}^{j}$	Time deviation of the trajectory *j* exit intersection
$p_{in}^{m}$	The last point outside the intersection whose coordinate is $\left(Lat_{in}^{m},Lng_{in}^{m}\right)$, point m in the trajectory *j*
$p_{in}^{s}$	The entry point of intersection side whose coordinate is $\left(Lat_{in}^{s},Lng_{in}^{s}\right)$
$p_{c}^{m+1}$	The inside point of intersection whose coordinate is $\left(Lat_{in}^{m+1},Lng_{in}^{m+1}\right)$, the point *m* + 1 in the trajectory *j*
$p_{out}^{s}$	The exit point of intersection side whose coordinate is $\left(Lat_{out}^{s},Lng_{out}^{s}\right)$
$p_{out}^{m+2}$	The first point outside the intersection whose coordinate is $\left(Lat_{out}^{m+2},Lng_{out}^{m+2}\right)$, the point *m* + 2 in the trajectory *j*
$N_{t}$	The number of the trajectories in terms of 5 min time slices, t=5min, 15min, 30min.

**Table 3:** The parameter definition of intersection traffic delay.

Parameter Notation	Definition
$N_{s}$	The number of stops of FCD
$N_{ns}$	The number of non-stops of FCD
$\tau_{i}$	The ratio of non-stop FCD to stop FCD in the direction *i* of the intersection
$v_{ij}$	The speed of the trajectory *j* passing through the intersection in the direction *i*
$r_{fj,\phi }$	The time stamp of the first point of the trajectory *j* in phase ϕ whose speed is 0 m/s.
$r_{lj,\phi }$	The time stamp of the last point of the trajectory *j* in phase ϕ whose speed is 0 m/s.
*n*	The number of the phases
$RT_{\phi }$	The red time of phases ϕ.
$g_{e,\phi }$	The effective green time in the phase ϕ, ϕ ranges from 1 to *n*.
*C*	A signal cycle
$\lambda_{\phi }$	The green ratio time in the phase ϕ, ϕ ranges from 1 to *n*.
$Q_{i}$	The traffic volume of the direction *i* of intersections
$\omega_{i}$	The flow ratio in direction *i*
$\eta_{i,\phi }$	The ratio between the flow ratio and the green signal ratio in the direction *i* of the phase ϕ.

**Table 4:** Internal evaluation parameters.

Parameter Notation	Southbound		Northbound		Eastbound		Westbound	
	Through	Left-Turn	Through	Left-Turn	Through	Left-Turn	Through	Left-Turn
Traffic Flow	328,692	17,348	237,894	19,728	18,065	20,047	16,388	12,347
Average velocity	3.20	2.39	3.19	2.11	2.64	2.53	3.13	2.63
Free Flow travel time	15	24	18	24	24	22	17	24
Average Delay time	52.61	64.22	50.60	81.88	57.53	62.53	64.79	49.09

Unit: Average velocity (m/s); Free travel time (s); Average Delay time (s).

**Table 5:** Number of stops and the ratio of the number of non-stop cars to the number of stop ones.

Parameter Notation	Northbound		Southbound		Eastbound		Westbound	
	Through	Left Turn	Through	Left Turn	Through	Left Turn	Through	Left Turn
*NOSF*	156,073	16,219	217,461	13,349	14,254	15,164	12,012	8441
*Total* $\tau_{i}$	0.81	0.10	0.79	0.16	0.74	0.66	0.68	0.76
*Evening peak* $\tau_{i}$	0.68	0.07	1.04	0.075	0.92	0.93	1.06	0.96
*Morning peak* $\tau_{i}$	1.15	0.088	0.90	0.073	0.74	0.80	0.36	0.70

**Table 6:** Relation between the green signal ratio and the flow ratio.

Parameter Notation	Northbound				Southbound				Eastbound				Westbound			
	Through		Left Turn		Through		Left Turn		Through		Left Turn		Through		Left Turn	
	Max	Min	Max	Min	Max	Min	Max	Min	Max	Min	Max	Min	Max	Min	Max	Min
$\omega_{i,\phi }$	0.421	0.271	0.039	0.012	0.581	0.404	0.033	0.014	0.039	0.015	0.040	0.016	0.032	0.006	0.029	0.010
$\eta_{i,\phi }$	1.064	0.740	0.120	0.030	2.131	1.015	0.083	0.044	0.191	0.047	0.099	0.039	0.178	0.016	0.075	0.029
	**Mean**	**Med**	**Mean**	**Med**	**Mean**	**Med**	**Mean**	**Med**	**Mean**	**Med**	**Mean**	**Med**	**Mean**	**Med**	**Mean**	**Med**
$\omega_{i,\phi }$	0.344	0.357	0.029	0.031	0.497	0.487	0.025	0.025	0.028	0.029	0.030	0.032	0.024	0.025	0.019	0.020
$\eta_{i,\phi }$	0.928	0.946	0.075	0.076	1.357	1.295	0.064	0.065	0.127	0.138	0.078	0.081	0.112	0.118	0.049	0.052
